# The Effect of Semiorganic Iodine-Containing Compounds on the Antibiotic Susceptibility of Pathogenic Microorganisms

**DOI:** 10.3390/biomedicines13081790

**Published:** 2025-07-22

**Authors:** Sabina T. Kenesheva, Seitzhan Turganbay, Ardak B. Jumagaziyeva, Gaukhar Askhatkyzy, Dana A. Askarova, Amir A. Azembayev, Alexandr I. Ilin, Oleg N. Reva, Tatyana A. Karpenyuk

**Affiliations:** 1 Scientific Center for Anti-Infectious Drugs, Almaty 050060, Kazakhstan; r_dawa@mail.ru (A.B.J.); askhatkyzyg@mail.ru (G.A.); askarova.dana08@gmail.com (D.A.A.); amirakan@mail.ru (A.A.A.); ilin_ai@mail.ru (A.I.I.); 2 Faculty of Biology and Biotechnology, Al-Farabi Kazakh National University, Almaty 050040, Kazakhstan; tatyana.karpenyuk@kaznu.kz; 3 Centre for Bioinformatics and Computational Biology, Department of Biochemistry, Genetics and Microbiology, University of Pretoria, Pretoria 0002, South Africa; oleg.reva@up.ac.za

**Keywords:** multidrug resistance, *E. coli*, *S. aureus*, antibiotics, iodine-containing compounds, amino acids

## Abstract

**Objectives:** The global rise in multidrug resistance underscores the urgent need for the development of novel and effective antimicrobial agents. Semi-organic iodine-containing complexes, owing to their unique properties, low likelihood of resistance development, and stability under various conditions, represent a promising avenue for the design of new therapeutic strategies. This study describes the synthesis of semi-organic iodine-containing complexes and the *in vitro* evaluation of their impact on antibiotic susceptibility modulation in the multidrug-resistant pathogenic microorganisms *S. aureus* and *E. coli*. **Methods:** The physicochemical properties of the semiorganic compounds were characterized using UV-Vis spectroscopy, potentiometric, and titrimetric methods. Evaluation of antimicrobial activity was obtained according to CLSI protocols. The impact of semiorganic compounds on the *in vitro* susceptibility of MDR strains was evaluated by the disk diffusion method. **Results:** This study evaluated the effects of iodine-containing complexes KC-270 and KC-271 on the antibiotic susceptibility of *Staphylococcus aureus* BAA-39 and *Escherichia coli* BAA-196. The most pronounced effect was observed with KC-270 applied during the lag phase, which enhanced the activity of several antibiotics and, in some cases, restored susceptibility. KC-271 exhibited a weaker and more limited impact. The findings suggest that KC-270 has potential as a modulator of antibiotic susceptibility, particularly when administered at early stages of bacterial growth. **Conclusions:** The results support the ability of amino acid-based iodine coordination compounds to influence the antibiotic susceptibility of pathogenic bacteria, highlighting their potential as adjuvant agents to improve the effectiveness of current antimicrobial therapies. However, although changes in susceptibility were detected, neither compound fully eliminated resistance in the multidrug-resistant strains, indicating the necessity for further research into their mechanisms of action and possible synergistic interactions with antibiotics.

## 1. Introduction

Restoring the antibacterial activity of clinically important antibiotics that have lost their efficacy due to reduced uptake by pathogens or the spread of specific resistance mechanisms is a major challenge in public health.

The 2024 WHO Bacterial Priority Pathogens List (BPPL) includes 24 pathogens across 15 families of antibiotic-resistant bacterial pathogens. Notably, this list includes Gram-negative bacteria resistant to last-resort antibiotics, multidrug-resistant *Mycobacterium tuberculosis*, and other highly resistant pathogens such as *Salmonella*, *Shigella*, *Neisseria gonorrhoeae*, *Pseudomonas aeruginosa*, and *Staphylococcus aureus* [1,2].

The resistance of microorganisms to antiseptics and antibiotics is driven by adaptive mechanisms regulated by chromosomal DNA and mobile genetic elements—plasmids and transposons. These elements can move within the genome and disseminate resistance genes through horizontal gene transfer. Major resistance mechanisms also include limited drug penetration through the cell wall, drug target modification, enzymatic drug inactivation, active efflux via transport systems, and biofilm formation. Consequently, the rise of antimicrobial resistance (AMR) significantly limits available therapeutic options, contributing to increased morbidity and mortality rates [3,4,5,6,7].

Bacterial resistance poses a serious global health threat, necessitating comprehensive and adaptable strategies to combat resistant bacteria. Although the discovery of new classes of antibiotics provides temporary relief, their introduction into clinical use accelerates the selection and propagation of pathogens resistant to these agents [8]. Therefore, the development of alternative non-antibiotic anti-infective agents represents a promising direction for controlling the emergence and spread of resistant pathogens.

To address the AMR crisis and search for novel antibiotics or alternatives, a number of global initiatives are currently underway at different stages of development [9].

One promising approach is the use of non-antibiotic compounds known as antibiotic adjuvants that target bacterial resistance mechanisms. These agents can be combined with outdated antibiotics to enhance therapeutic efficacy. In recent years, the field of antibiotic adjuvants has rapidly expanded, particularly with investigations into mechanisms beyond β-lactamase inhibition.

Potentiators—or antibiotic adjuvants—are non-antibiotic agents that enhance the activity of antibiotics by minimizing or directly inhibiting bacterial resistance mechanisms. They help restore or improve susceptibility in resistant pathogens, thereby increasing the efficacy of antibiotics against drug-resistant bacteria [10,11].

Despite the potential of adjuvant therapy in antibacterial strategies, this area remains underexplored. It is, therefore, essential to reconsider antibiotic monotherapy and investigate the use of non-antibiotic adjuvants to slow the development of antibiotic resistance.

Kumar and colleagues [12] studied a silver-guanidine derivative as a colistin potentiator against multidrug-resistant *Acinetobacter baumannii* and other colistin-resistant Gram-negative pathogens. The compound enhanced membrane disruption induced by colistin [12].

Liu et al. [13] identified phenylalanine-arginine-β-naphthylamide (PAbN), an efflux pump inhibitor, which enhanced the antimicrobial activity of the aminoglycoside neomycin against *Riemerella anatipestifer*, a pathogen in ducks. PAbN competes with neomycin for the same efflux system [13].

Xu and co-authors [14] investigated the synergistic action of colistin with naringenin, a flavanone subclass flavonoid, against colistin-resistant Gram-negative pathogens. This combination also inhibited biofilm formation and reduced bacterial load within the matrix. The antibacterial effect was attributed to increased membrane permeability caused by membrane destabilization, facilitating antibiotic uptake and bacterial killing [14].

Mirzaei et al. [15] studied the interaction of melittin, the principal component of bee venom, with vancomycin and rifampicin against multidrug-resistant *Staphylococcus epidermidis* (MDR-MRSE). They found that strains resistant to vancomycin and rifampicin became susceptible when treated in combination with melittin. However, the exact mechanisms underlying the synergy remain unclear [15].

Yuan and colleagues [16] proposed repurposing dimetridazole and ribavirin, previously approved for protozoal and viral infections, to reduce *Pseudomonas aeruginosa* virulence. In a *Caenorhabditis elegans* model, these agents were shown to reduce protease production and biofilm formation via quorum sensing modulation [16].

Iodine is well known for its broad-spectrum antimicrobial activity. Due to its accessibility, diverse biological functions, low cost, and environmental safety, iodine has high potential as an antimicrobial agent. To date, microbial resistance to iodine has not been reported, likely because iodine acts on multiple cellular targets. Its mechanism of action is based on oxidative properties that allow it to damage microbial cellular structures and disrupt protein and nucleic acid functions [17,18].

Iodine slows or completely inhibits protein synthesis in bacterial cells, breaks hydrogen bonds in proteins between amino groups of arginine and histidine and phenolic groups of tyrosine, and inactivates enzymes by oxidizing sulfur atoms in cysteine and methionine residues. It also disrupts electron transport, denatures DNA, and destabilizes membranes by oxidizing carbon–carbon double bonds in unsaturated fatty acids, ultimately leading to cell death. Iodine also interacts with nucleic acids by binding nucleotides, causing structural DNA/RNA damage, strand breaks, and mutations that impair replication and transcription [19,20].

However, the clinical use of elemental iodine is limited by its toxicity, volatility, and photodegradation under UV light. Aqueous iodine solutions are unstable and difficult to study due to halogenation reactions, a broad spectrum of organic compounds in solution, and the formation of random complexes.

To address these issues, efforts have focused on developing safer, more stable iodine-containing compounds that maintain efficacy under various conditions. The creation of stable iodine formulations has broadened iodine’s use in medicine, veterinary practice, and food industries. Iodine complexes with organic molecules—where iodine or iodide ions are coordinated to organic macromolecules—are more stable and less toxic than molecular iodine or potassium iodide solutions. These iodine complexes may contain both natural (e.g., chitosan, amino acids, albumin, starch, glycogen) and synthetic (e.g., polyvinyl alcohol, polyvinylpyrrolidone, polyamides) molecules [21,22,23].

Such complexation allows for controlled release, ensuring sustained antimicrobial activity with minimal cytotoxicity; enhanced stability, where iodine encapsulation protects it from degradation and preserves activity over time; improved adherence and reduced toxicity, minimizing exposure to high iodine concentrations for human cells and tissues.

Unlike traditional antiseptics such as povidone-iodine or potassium iodide, which primarily act through rapid, nonspecific oxidation of cellular components, coordination complexes containing amino acids and iodine appear to exert their effects in a more selective and moderate manner. Owing to the potential for cellular uptake and the biocompatibility of amino acid ligands, such complexes may modulate intracellular processes, including gene expression, metabolism, and ionic homeostasis. It is assumed that the amino acids incorporated into the complex play an active role not only in stabilizing the compound structurally but also in mediating its biological activity [24].

The aim of this study is to evaluate, *in vitro*, the effect of iodine-containing complexes with organic ligands on the antibiotic susceptibility of certain multidrug-resistant pathogenic bacterial strains.

## 2. Materials and Methods

### 2.1. Reagents

Lithium iodide (Sigma-Aldrich, St. Louis, MO, USA); potassium iodide (Sigma-Aldrich, St. Louis, MO, USA); cesium chloride (Sigma-Aldrich, St. Louis, MO, USA); iodine (Labpharm, JSC “Troitsky iodine plant”, Troitsk, Russia); glycine (Titan Biotech Ltd., Bhiwadi, Rajasthan, India); sodium chloride (Mikhaylovsky Chemical Reagent Plant, Malinovoye Ozero, Altai Territory, Russia); monobasic potassium phosphate (JSC “Base No. 1 Khimreaktivov”, Staraya Kupavna, Moscow Region, Russia); disodium phosphate dodecahydrate (CJSC “Kupavnareaktiv”, Staraya Kupavna, Moscow Region, Russia); sodium salt of resazurin (Sigma-Aldrich, St. Louis, MO, USA); trypticase soy agar (HiMedia Laboratories, Mumbai, Maharashtra, India); trypticase soy broth (HiMedia Laboratories, Mumbai, Maharashtra, India); Mueller–Hinton agar (HiMedia Laboratories, Mumbai, Maharashtra, India); Mueller–Hinton broth (HiMedia Laboratories, Mumbai, Maharashtra, India); ethanol (Talgar Spirt, Talgar, Kazakhstan).

Commercial antibiotic-impregnated discs (HiMedia Laboratories, Mumbai, Maharashtra, India) containing the required antimicrobial agents were used as antimicrobial agents.

The reagents used for the present work were of analytical grade, obtained from commercial sources, and used without further purification.

### 2.2. Iodine-Containing Semiorganic Compounds

The test compounds included original iodine-containing coordination compounds with amino acids, KC-270, KC-271, synthesized by the Laboratory of New Substances and Materials of JSC “Scientific Center for Anti-Infectious drugs” (Almaty, Kazakhstan).

### 2.3. Test Strains and Growth Conditions

The following MDR test strains were used in this study:

*Staphylococcus aureus* subsp. *aureus* (ATCC BAA-39)—a strain obtained from the American Type Culture Collection (ATCC). This strain belongs to SCCmec type III and carries several resistance-associated genes, including mecA (encoding the methicillin-binding protein), msrA1 (encoding an ATP-binding cassette efflux pump associated with antibiotic resistance), pls (a surface-associated methicillin resistance factor), and ccr (conferring resistance to non-β-lactam antibiotics).

*Escherichia coli* (ATCC BAA-196)—a strain obtained from the American Type Culture Collection (ATCC). It exhibits multidrug resistance mediated by a class A β-lactamase encoded by the TEM-10 plasmid.

Prior to the experiments, the test microbial cultures were reactivated following cryopreservation. Bacterial viability was evaluated by assessing the intensity of microbial growth on the corresponding elective agar-based nutrient medium after streak inoculation. Incubation was performed in a thermostat (Binder GmbH, Tuttlingen, Germany) for 18–24 h at 37 ± 1 °C. Microscopic examination (Leica Microsystems GmbH, Wetzlar, Germany) was subsequently conducted to confirm the purity of the test strains and verify the preservation of their morphological and cultural characteristics. Trypticase soy agar and broth were used as the culture media.

### 2.4. Synthesis of the Iodine-Containing Semiorganic Compounds with Amino Acids

To synthesize the KC-270 coordination compound, 0.9008 g (12.0 mmol) of glycine was dissolved in 10 mL of purified water in a 50 mL beaker. Glycine was chosen due to its structural simplicity, biocompatibility, and ability to form stable coordination bonds. Its minimal steric hindrance and metabolic relevance make it a suitable model ligand for preliminary studies.

The mixture was heated in a 40 °C water bath and stirred with a glass rod until the amino acid fully dissolved (Solution 1). Separately, 2.4093 g (18.0 mmol) of lithium iodide, 1.5228 g (6.0 mmol) of iodine, and 1.0102 g (6.0 mmol) of cesium chloride were finely powdered using an agate mortar and pestle and dissolved in 10 mL of purified water at room temperature (Solution 2). The molar ratios of glycine, lithium iodide, iodine, and cesium chloride were maintained at 2:3:1:1, with an excess of lithium iodide, iodine, and cesium chloride required for the complete reaction of glycine. The resulting solution, solution 2, was added to solution 1 in a 50 mL flask with a stopper. The reaction mixture was sealed and stirred at room temperature (RT) for 5 min, followed by 48 h of storage in the dark at RT to allow the system to reach equilibrium. Afterward, the mixture was heated in 50 °C water bath for 10 min and filtered under vacuum using a Schott filter into a crystallizer. The crystallizer was placed in a dark glass desiccator containing anhydrous calcium chloride as a desiccant to evaporate most of the water. The resulting crystals were collected under vacuum using a Schott filter, washed twice with ethanol, cooled to 0 °C, and further dried on an ashless black tape filter. The crystals were weighed, transferred to a flask with a glass stopper, and stored in a refrigerator. The final yield was 4.64 g (78%) of single crystals. The remaining KC-271 complex was synthesized using the same method described above, except that potassium iodide was used instead of lithium iodide.

### 2.5. UV–Vis Spectroscopy

UV-Vis spectra of the samples were collected using a LAMBDA-35 UV–Vis spectrophotometer (PerkinElmer, Waltham, MA, USA). The samples were dissolved in a water solution (1 mg/mL), and the solvent was used as a reference. The scanning range was 190–1100 nm.

### 2.6. Determination of the Iodine Content

The concentration of halogens (iodine) in the complexes was measured by sodium thiosulfate titration. The concentration of molecular iodine per 1000 g of the complex was measured by Equation (1):

(1) $$C_{I_{2}}=\frac{V_{1}\times K_{1}\times 12.69}{m}$$

where:

V_1_—volume of 0.05 M sodium thiosulfate spent for complete titration (mL);

K_1_—correction on sodium thiosulfate concentration in the buffer, for 0.05 M solution K1 = 0.5;

m—weight of the complex (g).

The concentration of KI was measured by Equation (2) using titration with silver nitrate:

(2) $$C_{\mathrm{KI}}=\frac{(V_{2}\times K_{2}-V_{1}\times K_{1})\times 16.59}{m}$$

where:

V_1_—volume of 0.05 M sodium thiosulfate spent for complete titration (mL);

V_2_—volume of 0.05 M AgNO_3_ spent for complete titration (mL);

K_1_ = K_2_ = 0.5;

m—weight of the complex (g).

### 2.7. Evaluation of the Antimicrobial Activity

The antimicrobial activity testing procedure was carried out using the broth microdilution method, as recommended by the Clinical and Laboratory Standards Institute [25,26,27]. Stock solutions of the coordination compounds were prepared according to Equation (3) at a 2× concentration.

(3) $$m(\mathrm{AA})=\frac{C\times V}{a},$$

where:

m (AA)—calculated mass of the test compound (mg);

C—target concentration of the agent (µg/mL);

V—volume of solvent required for the agent (mL);

a—potency of the agent (µg/mL).

A sterile isotonic sodium chloride solution was used as the solvent for the coordination compounds. Since the main active component of coordination compounds was free molecular iodine (I_2_), which interacts with oxidizable SH- and OH-groups of amino acid residues in proteins and peptides of nutrient media—thus reducing its intrinsic antimicrobial potential—a minimal nutrient medium was selected to adequately assess antimicrobial activity. A 4% Mueller–Hinton broth was used as the optimized cultivation medium for bacterial growth.

Antimicrobial activity was assessed by performing a series of two-fold dilutions. To the required number of wells in a 96-well plate, 150 µL of 4% Mueller–Hinton broth was added. Then, 150 µL of the corresponding coordination compound solution was added to the first well of each row. A series of two-fold serial dilutions were performed until the desired number of dilutions was achieved.

Into each well containing the 150 µL mixture (sample + broth), as well as into the positive control wells, 15 µL of the working microbial inoculum was added. The final bacterial concentration in each well after inoculation was approximately 1.5 × 10^5^ CFU/mL (DEN-1, Biosan, Riga, Latvia). The plates were incubated for 18–24 h at 37 ± 1 °C under aerobic conditions (Binder, Germany).

Following incubation, 25 µL of a 0.01% aqueous resazurin solution was added to each well. Plates were further incubated for 30 min to 4 h until a visible pink coloration appeared. Results were recorded colorimetrically (λ = 540 nm) based on microbial cell viability in the presence of various concentrations of the test samples (Multiskan Ascent, Thermo Fisher Scientific, Vantaa, Finland). The minimum inhibitory concentration (MIC) was defined as the lowest concentration of the antimicrobial agent that inhibited visible growth of the tested microorganism.

### 2.8. Determination of Bacterial Growth Curves

Growth curves of *Staphylococcus aureus* ATCC BAA-39 and *Escherichia coli* ATCC BAA-196 were obtained using a Multiskan Ascent microplate spectrophotometer (Multiskan Ascent, Thermo Fisher Scientific, Vantaa, Finland). Overnight broth cultures of the test strains were prepared in Mueller–Hinton broth and incubated at 37 °C. The next day, an aliquot of each overnight culture was transferred into fresh Mueller–Hinton broth and adjusted to 0.5 McFarland, corresponding to an approximate cell density of 1.5 × 10^8^ CFU/mL (DEN-1, Biosan, Riga, Latvia). A total of 150 μL of the standardized suspension was dispensed into the wells of a sterile 96-well microtiter plate. The plate was incubated at 37 °C for 18 h, and optical density at 600 nm (OD600) was measured at regular intervals to monitor bacterial growth.

### 2.9. Evaluation of the Effect of Iodine-Containing Compounds with Amino Acids on Antibiotic Susceptibility of Pathogenic Test Strains

#### 2.9.1. Preparation of Test Strain Suspensions

Suspensions of test microorganisms aged 18–24 h were used in this study. Cultivation was performed in Mueller–Hinton broth using a batch culture method at 37 ± 1 °C with continuous shaking in an incubator shaker at 100 rpm. The final bacterial concentration was adjusted to 1.5 × 10^8^ CFU/mL.

#### 2.9.2. Treatment of Test Strains with Iodine-Containing Compounds

To investigate non-lethal cellular responses without compromising bacterial viability, a concentration corresponding to ½ of the minimum inhibitory concentration (MIC) was used. This subinhibitory level is widely applied in antimicrobial research, as it allows the assessment of physiological and regulatory effects without inducing cell death or growth arrest [28].

The test strains were exposed to sublethal concentrations (½ MIC) of the coordination compounds. Untreated cultures receiving an equivalent volume of 0.9% sodium chloride solution served as negative controls. After reaching the lag phase and mid-log phase, the appropriate compounds were added to each group. The exposure time was 10 min, after which the cells were washed three times with phosphate-buffered saline (PBS) by centrifugation at 5000 rpm for 7 min.

#### 2.9.3. Evaluation of Changes in Antibiotic Susceptibility by Disc Diffusion Method

Changes in antibiotic susceptibility profiles were assessed using the disc diffusion method, following the protocol recommended by CLSI. A single colony of the test strain (after 18–24 h of incubation) was transferred into a sterile tube containing isotonic solution and adjusted to 0.5 McFarland standard (DEN-1, Biosan, Latvia), corresponding to 1.5 × 10^8^ CFU/mL. A sterile cotton swab was used to evenly inoculate Mueller–Hinton agar (Himedia, India) in three directions while rotating the Petri dish at 60°. Antibiotic discs (Himedia, India) were then placed on the inoculated agar using sterile forceps. The plates were incubated for 18–24 h at 37 ± 1 °C. After incubation, the diameter of the inhibition zones around each disc was measured. All experiments were performed in triplicate. Mean values and standard deviations were calculated to assess reproducibility and variation. Where applicable, statistical significance between control and treated groups was assessed using an unpaired two-tailed Student’s *t*-test (*p* < 0.05) in GraphPad Prism, version 6.0 (GraphPad Software, San Diego, CA, USA). However, the final interpretation of antibiotic susceptibility was based on the categorical zone diameter breakpoints defined by CLSI guidelines, regardless of statistical significance.

### 2.10. Use of Generative Artificial Intelligence

In this paper, generative artificial intelligence (ChatGPT, GPT-4o, OpenAI) was used for the partial analysis and visualization of selected data.

## 3. Results

### 3.1. Physicochemical Analysis of the Iodine-Containing Semiorganic Compounds with Amino Acids

The physicochemical properties, including color, percentage yield, melting point, and decomposition temperature of the synthesized coordination compounds, are summarized in Table 1. The KC-270 and KC-271 coordination compounds ranged in color from dark gray to dark brown and exhibited high solubility in water.

The water solubility of KC-270 and KC-271 was determined at 25 °C and is presented in Table 1. KC-270 exhibited a solubility of 0.1 g/mL, while KC-271 showed a significantly higher solubility of 2.0 g/mL. The increased solubility of KC-271 is attributed to the strong hydration effect of lithium iodide compared to potassium iodide in KC-270. The presence of cesium chloride had a minimal impact on the overall solubility, suggesting that lithium’s high hydration capacity plays a key role in solubility enhancement. However, they were insoluble in most organic solvents, including benzene, cyclohexane, chloroform, and o-xylene.

### 3.2. UV–Vis Spectral Analysis

**Figure 1:**
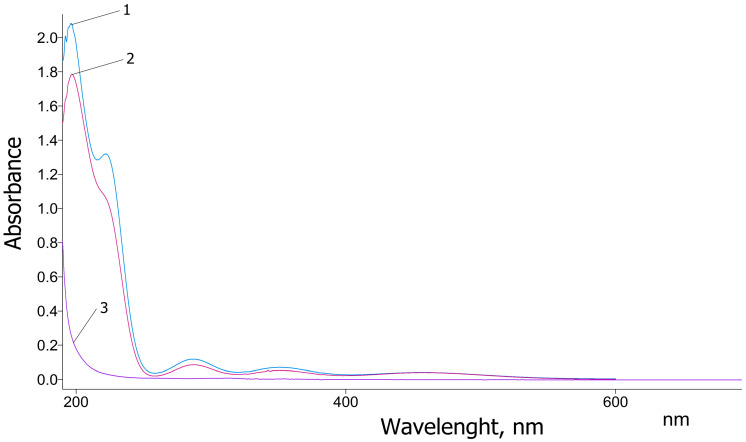
UV-Vis spectra of KC-270 (1), KC-271 (2), and glycine (3).

The UV–Vis spectra of pure glycine, as well as the newly synthesized coordination complexes KC-270 and KC-271, are presented in Figure 1. Pure glycine exhibits a strong absorption peak at 200 nm, which rapidly decreases and reaches a minimum around 240 nm. This absorption band is attributed to the π→π* electronic transition characteristic of glycine in the ultraviolet region, consistent with previously reported data [29]. In contrast, the UV–Vis spectra of the synthesized KC-270 and KC-271 complexes exhibit distinct absorption features, indicating significant changes in the electronic environment due to metal coordination. The newly observed peaks at 197 nm, 220 nm, 287 nm, and 351 nm suggest ligand-to-metal charge transfer (LMCT) interactions and alterations in molecular orbitals due to complexation. The value 197 nm—slightly shifted compared to the 200 nm peak of pure glycine, indicating molecular orbital modifications due to ligand–metal interactions. The value 220 nm—a newly observed peak, possibly resulting from altered electronic transitions within the ligand or charge redistribution effects caused by complex formation.

The values 287 nm and 351 nm—attributed to the presence of I_3_^−^, suggesting that triiodide species are formed within the coordination complex. These findings are consistent with the results reported by Sai et al. [30], confirming the successful complexation of glycine with iodine. The presence of the characteristic absorption peak of I_3_^−^ further supports the interaction between glycine and metal iodides, leading to the stabilization of triiodide species in the synthesized complexes.

### 3.3. Bacterial Growth Curves Results

**Figure 2:**
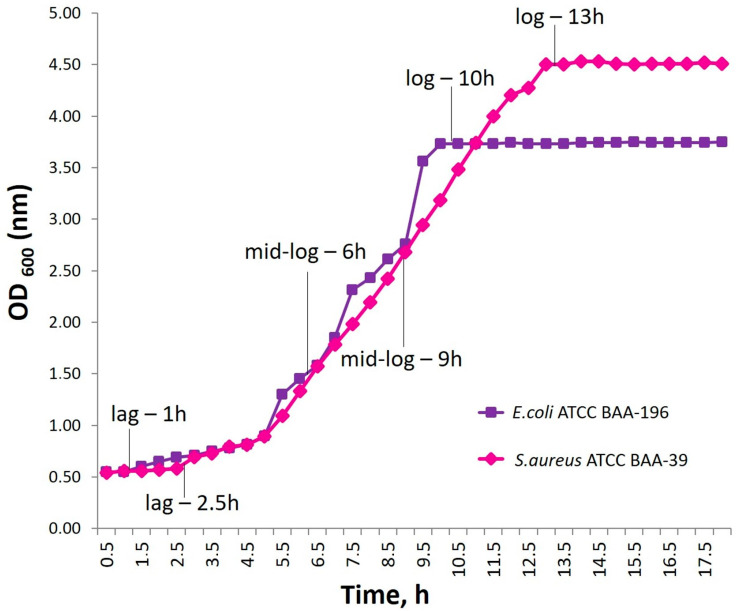
Growth curves of *S. aureus* ATCC BAA-39 and *E. coli* ATCC BAA-196 highlighting the durations of key growth phases used for experimental planning.

The results demonstrated that, for *S. aureus* ATCC BAA-39, the lag phase lasted approximately 2.5 h, the exponential phase ended by 13 h of incubation, and the mid-log point was reached at 9 h. For *E. coli* ATCC BAA-196, the lag phase lasted approximately 1 h, the exponential phase extended up to 10 h, and the mid-log point occurred at 6 h (Figure 2). Based on these data, the test compounds were added at the end of the lag phase and at the mid-logarithmic point of the exponential phase for subsequent treatment experiments.

### 3.4. The Effect of Iodine-Containing Compounds with Amino Acids on Antibiotic Susceptibility of Pathogenic Test Strains

The study assessing the impact of iodine coordination compounds with amino acids on changes in antibiotic susceptibility was conducted using multidrug-resistant reference strains of *Staphylococcus aureus* ATCC BAA-39 and *Escherichia coli* ATCC BAA-196.

The aim of this study was to evaluate the alteration in antibiotic susceptibility of multidrug-resistant microorganisms under the influence of iodine-containing coordination complexes.

In an *in vitro* assay of antimicrobial activity, it was found that the tested coordination compounds—KC-270 and KC-271—demonstrated efficacy against the reference strains *S. aureus* ATCC BAA-39 and *E. coli* ATCC BAA-196.

Subsequent experiments were performed using ½ of the minimum inhibitory concentration (MIC) of the coordination compounds, with MIC values presented in Table 2.

Aqueous solutions of the tested compounds were added to the microbial cultures at two growth stages: at the end of the lag phase and in the middle of the exponential growth phase (mid-log). These stages represent metabolically different states: the lag phase involves cellular adaptation and metabolic reprogramming, while the mid-log phase corresponds to active cell division and exponential growth. This approach allowed us to assess whether the modulatory effects on antibiotic susceptibility are influenced by the physiological state of the bacterial cells.

For *E. coli* BAA-196, the lag phase lasted 1 h, while, for *S. aureus* BAA-39, it was 2.5 h. The midpoint of the exponential phase was reached after 6 h of incubation for *E. coli* BAA-196 and after 9 h for *S. aureus* BAA-39.

In addition, a panel of antibiotics was selected to evaluate the potentiating activity of the coordination complexes. The selection included representatives of different antibiotic classes commonly used to treat infectious diseases, as well as antibiotics to which the test strains were known to be resistant.

MDR *Staphylococcus aureus*. Antimicrobial susceptibility testing was performed using 13 antibiotics from various classes:

- β-lactams: ampicillin, amoxicillin, carbenicillin, oxacillin, ceftriaxone, cefepime, cefazolin;
- Macrolides: azithromycin, erythromycin;
- Aminoglycosides: amikacin, gentamicin, kanamycin, tobramycin.

**Figure 3:**
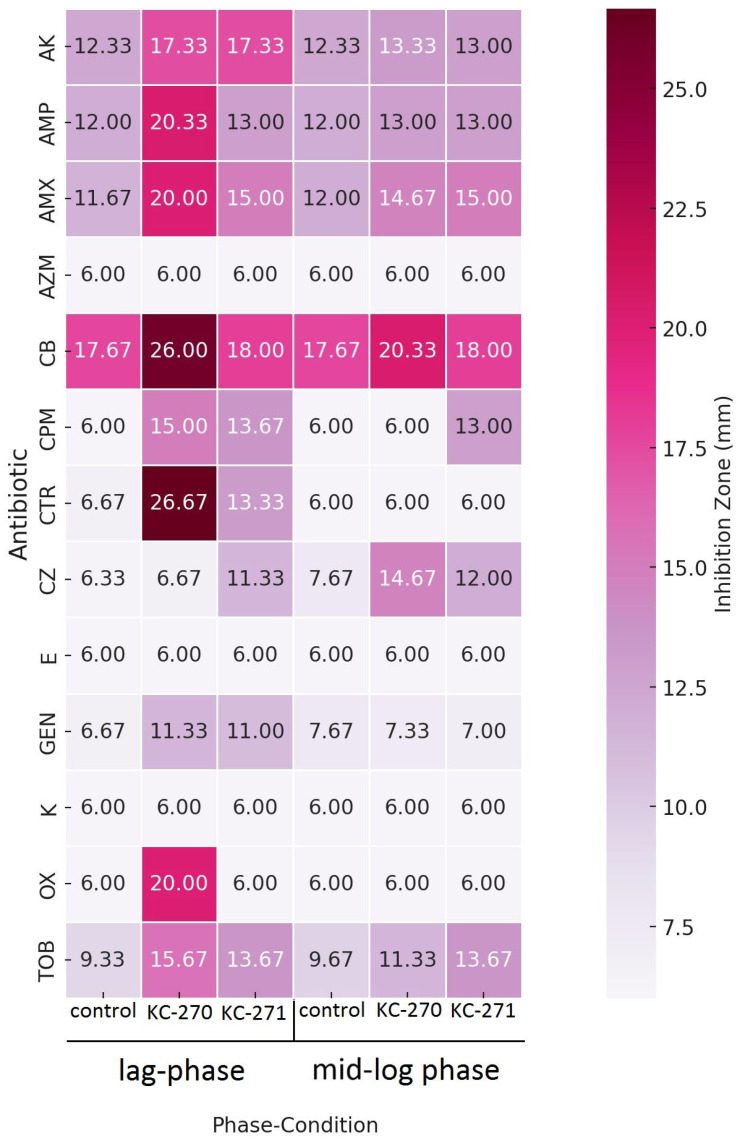
Results of antimicrobial susceptibility testing on *S.aureus* BAA-39 after treatment with KC-270 and KC-271; AK—amikacin; AZM—azithromycin; AMP—ampicillin; AMX—amoxicillin; CB—carbenicillin; CTR—ceftriaxone; CPM—cefepime; CZ—cefazolin; E—erythromycin; GEN—gentamicin; K—kanamycin; OX—oxacillin; TOB—tobramycin.

The results of antibiotic susceptibility testing following treatment of the strain with the iodine-containing compounds KC-270 and KC-271 are presented in Table 3 and Figure 3, reflecting outcomes at both growth stages.

According to the data, *S. aureus* BAA-39 exhibited susceptibility to amikacin, oxacillin, and ceftriaxone when treated with KC-270 during the lag phase. Additionally, a shift toward intermediate susceptibility was observed for cefepime and tobramycin. In contrast, exposure to KC-270 at the midpoint of the exponential phase did not result in detectable changes in antibiotic susceptibility.

A statistically significant increase in inhibition zone diameters was observed in the lag phase for ampicillin (12.00 ± 1.00 mm vs. 20.33 ± 0.58 mm, *p* = 0.00009), amoxicillin (11.67 ± 0.58 mm vs. 20.00 ± 0.00 mm, *p* = 0.0019), carbenicillin (17.67 ± 0.58 mm vs. 26.00 ± 1.73 mm, *p* = 0.00795), and gentamicin (6.67 ± 0.58 mm vs. 11.33 ± 1.15 mm, *p* = 0.00515); and in the mid-log phase for amoxicillin (12.00 ± 0.00 mm vs. 14.67 ± 0.58 mm, *p* = 0.00565), carbenicillin (17.67 ± 0.58 mm vs. 20.33 ± 0.58 mm, *p* = 0.00554), and cefazolin (7.67 ± 0.58 mm vs. 14.67 ± 0.58 mm, *p* = 0.00504).

However, the final interpretation of antibiotic susceptibility was based on the categorical zone diameter breakpoints defined by CLSI standards, regardless of statistical significance. Accordingly, despite the observed increases in zone diameters, the strain remained classified as resistant to these antibiotics. The remaining antimicrobial agents were ineffective against the test strain. Furthermore, treatment with KC-270 during the lag phase led to visible growth suppression across all experimental plates.

Treatment with the coordination compound KC-271, applied at the end of the lag phase, also affected the antibiotic susceptibility profile of *S. aureus* BAA-39. An increase in susceptibility to amikacin was observed (Table 3, Figure 3), whereas kanamycin, another representative of the aminoglycoside class, did not show a similar effect.

A statistically significant increase in inhibition zone diameters was observed in the lag phase following KC-271 treatment for amoxicillin (11.67 ± 0.58 mm vs. 15.00 ± 1.00 mm, *p* = 0.0164), ceftriaxone (6.67 ± 0.58 mm vs. 13.33 ± 1.53 mm, *p* = 0.0459), cefepime (6.00 ± 0.00 mm vs. 13.67 ± 1.15 mm, *p* = 0.0239), cefazolin (6.33 ± 0.58 mm vs. 11.33 ± 0.58 mm, *p* = 0.0011), gentamicin (6.67 ± 0.58 mm vs. 11.00 ± 0.00 mm, *p* = 0.0016), and tobramycin (9.33 ± 0.58 mm vs. 13.67 ± 1.15 mm, *p* = 0.0229).

In the mid-log phase, statistically significant increases were also recorded for amoxicillin (12.00 ± 0.00 mm vs. 15.00 ± 0.00 mm, *p* < 0.0001), cefepime (6.00 ± 0.00 mm vs. 13.00 ± 1.73 mm, *p* = 0.0067), cefazolin (7.67 ± 0.58 mm vs. 12.00 ± 0.58 mm, *p* = 0.00001), and tobramycin (9.67 ± 1.15 mm vs. 13.67 ± 1.53 mm, *p* = 0.0083).

Nonetheless, the final evaluation of antibiotic susceptibility adhered to the categorical zone diameter criteria established by CLSI, irrespective of statistical significance. As a result, all treated cultures remained classified as resistant.

Based on the obtained data, coordination compounds KC-270 and KC-271 induced measurable but subclinical modulation of antibiotic susceptibility and growth behavior in the *S. aureus* BAA-39 strain, with the most pronounced effects occurring during the lag phase.

MDR *Escherichia coli.* The antibiogram of *E. coli* BAA-196 following incubation with various iodine coordination compounds was obtained for eight antimicrobial agents: ampicillin, amoxicillin, carbenicillin, oxacillin (β-lactams); chloramphenicol (phenicols); gentamicin and tobramycin (aminoglycosides); erythromycin (macrolides).

**Figure 4:**
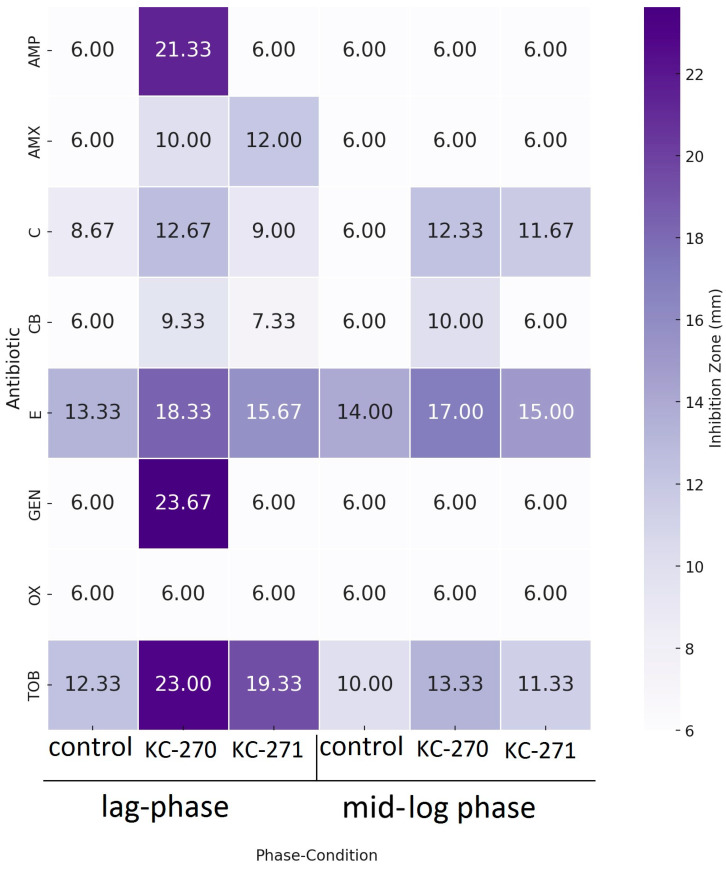
Results of antimicrobial susceptibility testing on *E. coli* BAA-196 after treatment with KC-270 and KC-271; AMP—ampicillin; AMX—amoxicillin; CB—carbenicillin; C—chloramphenicol; E—erythromycin; GEN—gentamicin; OX—oxacillin; TOB—tobramycin.

In this experiment, the *E. coli* BAA-196 strain exhibited a categorical shift to susceptibility for ampicillin, gentamicin, and tobramycin after treatment with KC-270 administered at the end of the lag phase (Table 4, Figure 4).

A statistically significant increase in inhibition zone diameters was observed following treatment with KC-270. In the lag phase, significant differences were recorded for amoxicillin (6.00 ± 0.00 mm vs. 10.00 ± 0.00 mm, *p* < 0.0001), carbenicillin (6.00 ± 0.00 mm vs. 9.33 ± 0.58 mm, *p* = 0.0076), chloramphenicol (8.67 ± 0.58 mm vs. 12.67 ± 0.58 mm, *p* = 0.0041), and erythromycin (13.33 ± 0.58 mm vs. 18.33 ± 0.58 mm, *p* = 0.0001).

In the mid-log phase, increases in zone diameters were also observed for carbenicillin (6.00 ± 0.00 mm vs. 10.00 ± 0.00 mm, *p* < 0.0001), chloramphenicol (6.00 ± 0.00 mm vs. 12.33 ± 0.58 mm, *p* = 0.0061), and tobramycin (10.00 ± 0.00 mm vs. 13.33 ± 1.15 mm, *p* = 0.0015).

Similarly, treatment with KC-271 resulted in statistically significant improvements in antimicrobial activity. In the lag phase, amoxicillin (6.00 ± 0.00 mm vs. 12.00 ± 0.00 mm, *p* < 0.0001) and tobramycin (12.33 ± 1.15 mm vs. 19.33 ± 0.58 mm, *p* = 0.0002) showed marked increases in inhibition zone diameters. In the mid-log phase, a significant increase was also recorded for chloramphenicol (6.00 ± 0.00 mm vs. 11.67 ± 0.58 mm, *p* = 0.0004).

Nonetheless, the final interpretation of antibiotic susceptibility was based on the categorical zone diameter breakpoints defined by CLSI standards, regardless of statistical significance. Consequently, the *E. coli* ATCC BAA-196 strain remained classified as resistant to all tested antibiotics (Table 4, Figure 4).

## 4. Discussion

This study focused on a Gram-positive strain, *Staphylococcus aureus* BAA-39, and a Gram-negative strain, *Escherichia coli* BAA-196. These bacterial strains are of particular interest due to their role as causative agents of acute infections and their broad-spectrum resistance to a wide range of antimicrobial agents.

The effects of sub-inhibitory concentrations of iodine-containing complexes were evaluated at two distinct bacterial growth phases—the lag phase and mid-logarithmic phase—without inducing cell death. The selected concentrations were introduced into bacterial cultures at these specific time points to determine their influence on antibiotic susceptibility.

In *S. aureus* BAA-39, treatment with KC-270 during the lag phase led to pronounced shifts in the antibiotic susceptibility profile. The strain exhibited a categorical shift to susceptibility for amikacin, oxacillin, and ceftriaxone. Statistically significant increases in growth inhibition zones were also observed for ampicillin, amoxicillin, carbenicillin, and gentamicin. Moreover, resistance to cefepime and tobramycin decreased to an intermediate level. In contrast, application of KC-270 during the mid-log phase resulted in more modest changes, with statistically significant but non-categorically shifting increases in inhibition zones for amoxicillin, carbenicillin, and cefazolin.

Treatment with the complex KC-271 also altered the antibiotic susceptibility of *S. aureus* BAA-39, although the effect was less pronounced compared to KC-270. The most notable result was the regained susceptibility to amikacin when the compound was introduced during the lag phase. Significant increases in inhibition zones were also recorded for gentamicin, amoxicillin, ceftriaxone, cefepime, cefazolin, and tobramycin. However, kanamycin, another aminoglycoside, showed no change. At the mid-log phase, KC-271 induced significant increases for amoxicillin, cefepime, cefazolin, and tobramycin only. Despite these statistically significant enhancements, CLSI-defined categorical interpretations did not indicate a reclassification to susceptibility for most antibiotics, and the strain retained its multidrug-resistant phenotype.

In *E. coli* BAA-196, KC-270 showed a stronger modulatory effect. When administered during the lag phase, the strain demonstrated a categorical shift to susceptibility to ampicillin, gentamicin, and tobramycin. Additionally, statistically significant enlargement of inhibition zones was observed for amoxicillin, carbenicillin, chloramphenicol, and erythromycin. When KC-270 was applied during the mid-log phase, increased zone diameters were recorded for carbenicillin, chloramphenicol, and tobramycin. Treatment with KC-271 led to a partial modulation of susceptibility in *E. coli*. During the lag phase, significant increases were observed for amoxicillin and tobramycin, although no categorical shifts in resistance were registered. In the mid-log phase, enhanced activity was recorded for chloramphenicol only. Despite some measurable effects, KC-271 did not lead to a reversal of the multidrug-resistant phenotype in this strain.

The findings indicate that both KS- KC-270 and KC-271 can modulate the antibiotic susceptibility of *S. aureus* BAA-39 and *E. coli* BAA-196. KC-270 was particularly effective when applied during the lag phase, enhancing the activity of several antibiotics and, in some cases, inducing susceptibility to previously ineffective agents. This may be attributable to the physiological state of bacteria during the lag phase, characterized by intense protein synthesis, increased nucleic acid content (especially RNA), and activation of various enzymatic pathways. Iodine, upon entering the bacterial cells, can interact with critical biomolecules, such as proteins, nucleotides, and fatty acids, possibly mediating these effects. Although both KC-270 and KC-271 are glycine-based iodine coordination compounds, they differ in their iodine content: 45.93% and 39.29% (*w/w*), respectively. This difference in active iodine concentration may partially explain the compound-specific effects observed in antibiotic sensitization assays.

The differential effects of iodine-containing coordination compounds observed between *S. aureus* and *E. coli* may, in part, be attributed to the fundamental structural differences between Gram-positive and Gram-negative bacteria. Gram-negative bacteria, such as *E. coli*, possess an outer membrane enriched with lipopolysaccharides and selective porins, which limits the penetration of many antimicrobial agents. Additionally, efflux pumps spanning the envelope can actively expel toxic compounds, reducing intracellular accumulation. In contrast, *S. aureus*, a Gram-positive organism, lacks an outer membrane and relies on a thick peptidoglycan layer, which is relatively more permeable to external agents. These structural distinctions likely influence the permeability and intracellular access of the coordination compounds, contributing to the observed variation in their modulatory effects on antibiotic susceptibility [31,32].

This study aimed to assess the potential of semi-organic iodine-containing coordination complexes to modulate the antibiotic susceptibility of resistant bacterial strains following exposure and subsequent washing of the cells. The results we obtained confirm that an effect on antibiotic susceptibility can be observed. However, at this stage of the research, we do not address the underlying mechanism of action, which remains to be elucidated.

It is reasonable to hypothesize that the mechanism of action of iodine-containing coordination complexes is multifactorial due to their composite structure and is directed toward multiple cellular targets. It is assumed that their antimicrobial effects may be mediated through a combination of mechanisms affecting both structural components of the bacterial cell envelope and regulatory elements of cellular metabolism and gene expression.

Our previous studies on gene expression in *Escherichia coli* and *Staphylococcus aureus* exposed to amino acid-based iodine complexes revealed that the primary targets were the bacterial cell wall and membrane. These structures underwent oxidative damage, resulting in increased permeability to metal ions, toxic compounds, and antibiotics. Such membrane disruption led to osmotic stress in both *E. coli* and *S. aureus*. This stress was accompanied by a rise in intracellular reactive oxygen species, triggering oxidative stress, damage to intracellular components, and activation of genes involved in oxidative stress protection. These processes collectively reduced bacterial virulence and antibiotic resistance [33].

In *S. aureus*, iodine treatment resulted in downregulation of TCA cycle genes (icd, sucC) and activation of glycolytic genes (pgk, gpmI, tpiA), indicating a shift toward anaerobic metabolism. Simultaneous upregulation of cydA, involved in microaerobic respiration, and suppression of the anaerobic enzyme pflB, pointed to a complex respiratory adjustment. Genes associated with amino acid biosynthesis were generally downregulated. Upregulation of menD and menH indicated restructuring of the electron transport chain. Increased expression of nirB, narGH, and fnt suggested a partial switch to nitrate and nitrite respiration. Lipid metabolism was also altered: genes for β-oxidation (fadA, fadB) were suppressed, while biosynthetic genes like accD were activated, likely reflecting membrane remodeling. Cell wall biosynthesis genes (atl, dacB, murD) and acetate kinase ackA were also upregulated, supporting acetyl-CoA production and wall reconstruction. DNA repair was carried out via ruvB, mutSL, and nth, whereas SOS response genes (recA, lexA) were suppressed. Osmoprotection was enhanced through upregulation of betB, opuD2, and potA, along with genes involved in ion transport (iron, potassium, sulfate), contributing to homeostasis under cell wall damage conditions [33,34].

In *E. coli*, exposure to iodine-containing complexes led to the downregulation of tricarboxylic acid (TCA) cycle genes (fumA, sdhA, sdhB, sucA–C) and glyoxylate shunt genes (acnB, gltA), along with the upregulation of glycolytic genes pfkA and tpiA. There was also increased expression of ndh and the DmsABC operon and suppression of narG, indicating a shift from aerobic to anaerobic catabolism. The complexes also suppressed genes involved in the transport of carbohydrates, fatty acids, and respiratory substrates (actP, mglABC, malE), as well as components of electron transport chains. At the same time, anabolic pathways were activated, including those for fatty acid and lipid biosynthesis, ribosomal protein production, and cell wall synthesis. Increased expression of spoT, argS, and glnS likely reflects an attempt by the cells to maintain metabolic activity under stress conditions. CopA and CueO genes played a key role in adaptation to osmotic stress and heavy metal detoxification [33,34,35].

Treatment with iodine-containing coordination compounds enhanced the expression of heavy metal efflux systems (copA, cueO, zitB) and detoxification enzymes in both *E. coli* and *S. aureus*, suggesting that these genes may serve as molecular markers of iodine-induced cell wall damage. In both species, suppression of organic solute transporters was observed, possibly representing a protective mechanism to limit intracellular iodine accumulation. Key shared responses in *E. coli* and *S. aureus* included downregulation of TCA cycle genes, enhanced biosynthesis of fatty acids and proteins, activation of redox homeostasis systems, and a shift toward anaerobic metabolism. Such metabolic reprogramming likely reduces virulence, weakens antibiotic resistance, and mitigates iodine-induced oxidative stress [33,34,35,36].

The antimicrobial action of iodine within coordination complexes is likely driven by its oxidative activity, which disrupts cellular integrity and interferes with the function of proteins and DNA.

The role of the organic ligand—specifically glycine—in these semi-organic complexes warrants particular attention. According to the literature, amino acids are capable of indirectly regulating gene expression, influencing translation initiation, signaling pathways, and mRNA selectivity. While such studies primarily focus on eukaryotic systems, evidence from *E. coli* confirms that amino acid supplementation causes significant metabolic shifts, including repression of endogenous amino acid synthesis, increased lipogenesis, and altered expression of energy metabolism-related genes. In eukaryotes, essential amino acids regulate a wide array of gene expression processes, including modulation of proteins involved in mRNA translation through eIF2α kinase (mGCN2). By altering the activity of translation initiation and elongation factors, amino acids can globally regulate protein synthesis and selectively enhance the translation of specific transcripts [37,38].

In bacteria, cis-regulatory elements known as riboswitches have been identified within the 5′ untranslated regions (UTRs) of mRNAs. These elements recognize a broad range of intracellular metabolites, including amino acids. Aptamer domains within riboswitches bind small molecules and modulate downstream expression platforms. Glycine riboswitches commonly regulate genes involved in glycine degradation pathways but have also been found upstream of genes related to glycine biosynthesis, transport, and conversion. These regulatory elements are widespread and have been identified in both Gram-positive and Gram-negative bacteria [39,40,41].

Semi-organic iodine compounds that incorporate glycine as the organic moiety may act as glycine analogs and potentially serve as ligands for glycine riboswitches. Targeting these riboswitches with glycine analogs may represent a novel antimicrobial therapeutic strategy. Inhibitors that mimic natural ligands—such as glycine analogs—can bind riboswitches and cause premature transcription termination, thereby suppressing the expression of key bacterial genes without directly interacting with proteins.

Although only a few potentiators, such as β-lactamase inhibitors, are currently used in clinical practice, other promising targets include resistance enzymes, efflux pumps, transcription factors regulating resistance genes, and systems maintaining the stability of resistance-associated mobile genetic elements.

Due to the physicochemical properties of iodine, these complexes are capable of inhibiting efflux systems by binding to pump proteins, disrupting membrane integrity, and enhancing permeability through oxidation of the lipid bilayer and interaction with outer membrane proteins; inducing oxidative stress with the destabilization of proteins through interaction with antibiotic-modifying or DNA repair enzymes. Moreover, the amino acids in semi-organic iodine compounds may exert regulatory effects, leading to metabolic overload, disruption of amino acid homeostasis, and altered gene expression in microbial cells.

While the exact reasons for the differential impact on individual antibiotics remain to be fully elucidated, the observed heterogeneity likely reflects the multifactorial nature of antibiotic action, bacterial resistance mechanisms, and cellular responses to iodine exposure. The iodine-containing compounds may alter membrane permeability, stress response pathways, or regulatory systems, thereby enhancing the activity of certain antibiotics depending on their molecular targets, uptake pathways, and stability against resistance factors. Importantly, the effects were not uniform across all antibiotics or growth phases, indicating that sensitization may be context-dependent and mediated by complex physiological interactions. Further research is needed to clarify these relationships.

## 5. Conclusions

Currently, the treatment of infectious diseases is becoming increasingly challenging due to the growing diversity of antibiotic- and antiseptic-resistant strains of pathogenic microorganisms. According to the World Health Organization (WHO), antimicrobial resistance (AMR) represents a global and serious threat to human health and development. The issue of pathogen resistance to conventional antibiotics is emerging as a key concern from both medical and socio-economic perspectives [42].

Iodine-containing coordination compounds incorporating amino acid ligands represent a promising class of substances that combine the antimicrobial properties of iodine with the bioregulatory potential of amino acids. The proposed mechanism of action involves inhibition of efflux pump systems, disruption of membrane architecture, induction of oxidative stress, and modulation of gene expression, thereby positioning these compounds as promising agents in the fight against antibiotic resistance [43].

The findings of this study demonstrate that the iodine coordination complexes KC-270 and KC-271 influence the antibiotic susceptibility profiles of both Gram-positive and Gram-negative bacteria. However, the extent and direction of this effect depend on the bacterial growth phase during treatment. This approach offers a valuable addition to current therapeutic options in the fight against multidrug-resistant (MDR) bacterial infections.

The development of broad-spectrum adjuvants may prove to be a more economically viable and therapeutically advantageous direction for contemporary medicine compared to the discovery of new antibiotics. The data confirm the potential of iodine coordination compounds with amino acids, suggesting their promise as adjuvant candidates to enhance the efficacy of existing antimicrobial agents.

The present study serves as a pilot investigation into the capacity of iodine-containing coordination compounds to modulate antibiotic susceptibility in multidrug-resistant bacterial strains. While compound-specific and growth phase-dependent changes were observed, the underlying mechanisms were not explored in this study and warrant further investigation.

## Figures and Tables

**Table 1:** Physicochemical properties of the KC-270 and KC-271 iodine-containing compounds.

Name of Indicators	Results	
	KC-270	KC-271
Empirical formula	C_4_H_10_I_3_KN_2_O_4_⋅CsCl	C_4_H_10_I_3_Li N_2_O_4_⋅CsCl
Molecular Weight, g/mol	739	708
pH	4.84	4.85
Solubility in water, g/mL	0.1 g/mL (at 25 °C)	0.2 g/mL (at 25 °C)
Melting temperature (°C)	188 (143–145)	162 (128–134)
Colour	Dark gray	Dark brown
Iodine content, (g/kg)	459.34 (45.93% (*w*/*w*))	392.90 (39.29% (*w*/*w*))
Iodide content	244.70	336.00
Yield (%)	78	75

**Table 2:** Antimicrobial activity of iodine-containing compounds with amino acids.

Iodine-Containing Compound	Test Strain	MIC Values, μg/mL (Based on I_2_ Concentration)
KC-270	*E. coli* BAA-196	1309.1
	*S. aureus* BAA-39	1309.1
KC-271	*E. coli* BAA-196	756.3
	*S. aureus* BAA-39	756.3

**Table 3:** Antibiotic susceptibility assessment results for *S. aureus* BAA-39 following treatment with iodine-containing compounds KC-270 and KC-271.

**Lag Phase**			
**Antibiotic, µg/Disc**	**Growth Inhibition zone, M ± SD, mm**		
	**Experiment**		**Control**
	**KC-270**	**KC-271**	
AK (10)	17.33 ± 0.58 **(S)**	17.33 ± 0.58 **(S)**	12.33 ± 0.58 **(R)**
AZM (30)	6.00 ± 0.00 **(R)**	6.00 ± 0.00** (R)**	6.00 ± 0.00 **(R)**
AMP (10)	20.33 ± 0.58 (R) *	13.00 ± 1.00 **(R)**	12.00 ± 1.00 **(R)**
AMX (10)	20.00 ± 0.00 (R) *	15.00 ± 1.00 (R) *	11.67 ± 0.58 **(R)**
CB (100)	26.00 ± 1.73 (-) *	18.00 ± 1.73 **(-)**	17.67 ± 0.58 **(-)**
CTR (30)	26.67 ± 0.58 **(S)**	13.33 ± 1.53 (R) *	6.67 ± 0.58 **(R)**
CPM (30)	15.00 ± 0.58 **(I)**	13.67 ± 1.15 (R) *	6.00 ± 0.00 **(R)**
CZ (30)	6.67 ± 0.58 **(R)**	11.33 ± 0.58 (R) *	6.33 ± 0.58 **(R)**
E (10)	6.00 ± 0.00 **(R)**	6.00 ± 0.00** (R)**	6.00 ± 0.00 **(R)**
GEN (30)	11.33 ± 1.15 (R) *	11.00 ± 0.00 (R) *	6.67 ± 0.58 **(R)**
K (30)	6.00 ± 0.00 **(R)**	6.00 ± 0.00** (R)**	6.00 ± 0.00 **(R)**
OX (1)	20.00 ± 0.00 **(S)**	6.00 ± 0.00** (R)**	6.00 ± 0.00 **(R)**
TOB (30)	15.67 ± 0.58 **(I)**	13.67 ± 1.15 (R) *	9.33 ± 0.58 **(R)**
**Mid-log phase**			
**Antibiotic, µg/disc**	**Growth inhibition zone, M** ** ± SD, mm**		
	**Experiment**		**Control**
	**KC-270**	**KC-271**	
AK (10)	13.33 ± 1.53 **(R)**	13.00 ± 0.00 **(R)**	12.33 ± 0.58 **(R)**
AZM (30)	6.00 ± 0.00 **(R)**	6.00 ± 0.00** (R)**	6.00 ± 0.00 **(R)**
AMP (10)	13.00 ± 1.00 **(R)**	13.00 ± 1.00 **(R)**	12.00 ± 0.00 **(R)**
AMX (10)	14.67 ± 0.58 (R) *	15.00 ± 0.00 (R) *	12.00 ± 0.00 **(R)**
CB (100)	20.33 ± 0.58 (-) *	18.00 ± 0.00 **(-)**	17.67 ± 0.58 **(R)**
CTR (30)	6.00 ± 0.00 **(R)**	6.00 ± 0.00** (R)**	6.00 ± 0.00 **(R)**
CPM (30)	6.00 ± 0.00 **(R)**	13.00 ± 1.73 (R) *	6.00 ± 0.00 **(R)**
CZ (30)	14.67 ± 0.58 (R) *	12.00 ± 0.58 (R) *	7.67 ± 0.58 **(R)**
E (10)	6.00 ± 0.00 **(R)**	6.00 ± 0.00** (R)**	6.00 ± 0.00 **(R)**
GEN (30)	7.33 ± 0.58 **(R)**	7.00 ± 0.00** (R)**	7.67 ± 0.58 **(R)**
K (30)	6.00 ± 0.00 **(R)**	6.00 ± 0.00** (R)**	6.00 ± 0.00 **(R)**
OX (1)	6.00 ± 0.00 **(R)**	6.00 ± 0.00** (R)**	6.00 ± 0.00 **(R)**
TOB (30)	11.33 ± 0.58 **(R)**	13.67 ± 1.53 (R) *	9.67 ± 1.15 **(R)**
Susceptibility		Intermediate resistance	Statistically significant difference

AK—amikacin; AZM—azithromycin; AMP—ampicillin; AMX—amoxicillin; CB—carbenicillin; CTR—ceftriaxone; CPM—cefepime; CZ—cefazolin; E—erythromycin; GEN—gentamicin; K—kanamycin; OX—oxacillin; TOB—tobramycin; (S)—culture is susceptible to the antimicrobial agent; (I)—culture exhibits intermediate resistance to the antimicrobial agent; (R)—culture is resistant to the antimicrobial agent; *—statistically significant difference; (-)—susceptibility interpretation not available.

**Table 4:** Antibiotic susceptibility assessment results for *E. coli* ATCC BAA-196 following treatment with iodine-containing compounds KC-270 and KC-271.

**Lag Phase**			
**Antibiotic, µg/disc**	**Growth Inhibition Zone, M ± SD, mm**		
	**Experiment**		**Control**
	**KC-270**	**KC-271**	
AMP (10)	21.33 ± 0.58 **(S)**	6.00 ± 0.00 **(R)**	6.00 ± 0.00 **(R)**
AMX (10)	10.00 ± 0.00 (R) *	12.00 ± 0.00 (R) *	6.00 ± 0.00 **(R)**
CB (100)	9.33 ± 0.58 (R) *	7.33 ± 0.58 **(R)**	6.00 ± 0.00 **(R)**
C (30)	12.67 ± 0.58 (R) *	9.00 ± 0.00 **(R)**	8.67 ± 0.58 **(R)**
E (10)	18.33 ± 0.58 (R) *	15.67 ± 0.58 **(R)**	13.33 ± 0.58 **(R)**
GEN (10)	23.67 ± 0.58 **(S)**	6.00 ± 0.00 **(R)**	6.00 ± 0.00 **(R)**
OX (1)	6.00 ± 0.00 **(-)**	6.00 ± 0.00 **(-)**	6.00 ± 0.00 **(-)**
TOB (30)	23.00 ± 0.00 **(S)**	19.33 ± 0.58 (R) *	12.33 ± 1.15 **(R)**
**Mid-log phase**			
**Antibiotic, µg/disc**	**Growth inhibition zone, M** ** ± ** **SD, mm**		
	**Experiment**		**Control**
	**KC-270**	**KC-271**	
AMP (10)	6.00 ± 0.00 **(R)**	6.00 ± 0.00 **(R)**	6.00 ± 0.00 **(R)**
AMX (10)	6.00 ± 0.00 **(R)**	6.00 ± 0.00 **(R)**	6.00 ± 0.00 **(R)**
CB (100)	10.00 ± 0.00 (R) *	6.00 ± 0.00 **(R)**	6.00 ± 0.00 **(R)**
C (30)	12.33 ± 0.58 (R) *	11.67 ± 0.58 (R) *	6.00 ± 0.00 **(R)**
E (10)	17.00 ± 1.00 **(R)**	15.00 ± 0.00 **(R)**	14.00 ± 0.00 **(R)**
GEN (10)	6.00 ± 0.00 **(R)**	6.00 ± 0.00 **(R)**	6.00 ± 0.00 **(R)**
OX (1)	6.00 ± 0.00 **(-)**	6.00 ± 0.00 **(-)**	6.00 ± 0.00 **(-)**
TOB (30)	13.33 ± 1.15 (R) *	11.33 ± 1.15 **(R)**	10.00 ± 0.00 **(R)**
Susceptibility		Intermediate resistance	Statistically significant difference

AMP—ampicillin; AMX—amoxicillin; CB—carbenicillin; C—chloramphenicol; E—erythromycin; GEN —gentamicin; OX—oxacillin; TOB—tobramycin; (S)—culture is susceptible to the antimicrobial agent; (I)—culture exhibits intermediate resistance to the antimicrobial agent; (R)—culture is resistant to the antimicrobial agent; *—statistically significant difference; (-)—susceptibility interpretation not available.

## Data Availability

The original contributions presented in this study are included in the article. Further inquiries can be directed to the corresponding authors.

## Abbreviations

The following abbreviations are used in this manuscript:

ATCC	American Type Culture Collection
BPPL	Bacterial Priority Pathogens List
CFU	Colony Forming Units
MDR	Multidrug Resistant
MIC	Minimum Inhibitory Concentration
WHO	World Health Organization
